# Age-dependent co-dependency structure of biomarkers in the general population of the United States

**DOI:** 10.18632/aging.101842

**Published:** 2019-02-28

**Authors:** Alan Le Goallec, Chirag J. Patel

**Affiliations:** 1 Department of Systems Biology, Harvard Medical School, Boston, MA 02115, USA; 2 Department of Biomedical Informatics, Harvard Medical School, Boston, MA 02115, USA

**Keywords:** aging, biomarkers, correlations, machine learning, demographics

## Abstract

Phenotypic biomarkers (e.g. cholesterol, weight, and glucose) are important to diagnose and treat diseases associated with aging. However, while many biomarkers are co-dependent (e.g. glycohemoglobin and glucose), it is generally unknown how age influences their co-dependence. In the following, we analyzed 50 biomarkers in 27,508 National Health and Nutrition Examination Survey (NHANES) participants (age range: 20 to 80, mean age: 46.3 years old, sexes: 48.9% males, 51.1% females, ethnicities: 46.0% Whites, 27.8% Hispanics, 20.0% non-Hispanic Blacks, 6.1% others) to investigate how the co-dependency structure of common biomarkers evolves with age and whether differences exist between sexes and ethnicities. First, we associated the change in correlations between biomarkers with chronological age. We identified six trends and replicated our top finding (height vs. systolic blood pressure) in participants of the UK Biobank (N=470,895). We found that, on average, correlations tend to decrease with age. Secondly, we examined how biomarkers predict other biomarkers in participants of different age groups. We found 17 (34%) biomarkers whose predictability decreases with age and 5 (10%) biomarkers whose predictability increases with age. A limitation of this study is that it cannot distinguish between biological changes related to aging and generational effects. Our results can be interactively explored here: http://apps.chiragjpgroup.org/Aging_Biomarkers_Co-Dependencies/.

## Introduction

Human biomarkers such as blood biomarkers (e.g. glucose, LDL-cholesterol, HDL-cholesterol, albumin) and anthropomorphic measures (e.g. blood pressure, BMI (body mass index), arm circumference) are of paramount importance for medicine and biomedical research, as they are used to diagnose disease, evaluate treatment, predict a clinical outcome, and even serve as proxy for clinical endpoints [1]. These biomarkers change markedly with age [2–6]. Further still, it is hypothesized that these biomarkers can predict age [6–9]. However, it is less understood how, in fact, the inter-dependencies of biomarkers themselves change with age.

Investigation of the co-dependency of biomarkers may shine light on shared etiology between diseases and biomarkers of disease. For example, in human genetics, investigators have been elucidating the shared “genetic architecture” between complex biomarkers, such as gene expression [10] and clinical biomarkers and disease [11]. Alongside genetics, age may play an influential role in the co-dependency of biomarkers. However, there has been to our knowledge no investigation to characterize the global correlation architecture of biomarkers, and how this architecture is affected by age.

**Figure 1:**
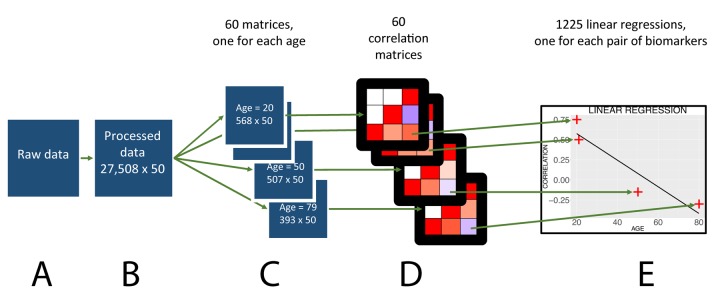
Flowchart to analyze the age trajectories of the correlations.

Here, we leverage an unhospitalized and US representative survey, called the National Health and Nutrition Examination Survey (NHANES), to investigate the correlation architecture between 50 biomarkers (a total of 1,225 pairwise correlations) in 27,508 participants (ages 20-80). Specifically, we investigated the co-dependency architecture of biomarkers using three different approaches. First, we hypothesized that the correlation structure of biomarkers changes with age. To address this primary hypothesis, we implemented a computational approach to test how correlations between biomarkers fluctuate between each age year between 20 through 80 (Figure 1). Second, we hypothesized that the biomarkers’ predictability -- or the variance explained between biomarkers -- also changes with age. To address this hypothesis, we estimated the predictability of each biomarker at each age year by building a regularized linear model to predict its value using all the other biomarkers as predictors. In this paper, we refer to “predictabilities” when discussing the prediction accuracies (R^2^ values) obtained on the 50 biomarkers. Third, we hypothesized that the predictor biomarkers selected by the models when predicting each target biomarker change with age. To test this hypothesis, we tracked changes of the regression coefficients of the elastic nets. For the three aforementioned hypotheses, we also tested for differences between both sexes and ethnicities. In summary, we found that age, along with sex and ethnicity, plays a major role in influencing biomarker co-dependency architecture. Understanding the age-dependent architecture will be important for future investigations in aging and age prediction [12].

A limitation of this paper is that NHANES is a cross-sectional survey. Accordingly, our findings do not distinguish between biological changes driven by aging at the individual level, and generational effects at the population level. When we use the terms “age trajectories” and “rates” in this paper, we are describing differences between the values computed on samples of different ages without implying that those differences are driven by aging.

## RESULTS

### National Health and Nutrition Examination Survey (NHANES) participants’ biomarkers are significantly correlated with age

**Figure 2:**
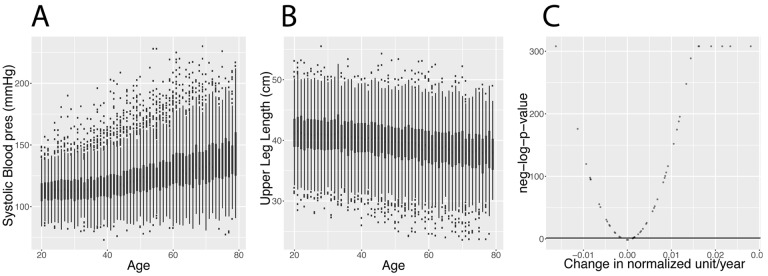
**Changes in biomarkers with age.** (**A**) Systolic blood pressure. Example of a biomarker that increases with age. (**B)** Upper leg length. Example of a biomarker that significantly decreases with age. (See discussion for explanation about the generational effect). (**C**) Volcano plot of the significance of the changes associated with age for the 50 biomarkers. The black horizontal line corresponds to the 0.05 significant threshold, after Bonferroni correction.

We analyzed 50 clinical biomarkers on 27,508 participant samples from the National Health and Nutrition Examination Survey (NHANES), a dataset constituted of non-institutionalized individuals [13] aged between 20 years old and up to 80 years old (excluded), with a mean age of 46.3 years old (Tables 1 and 2). The distribution of the ages can be found in Figure S1 (quantiles: 0%:20, 25%:32, 50%:45, 75%:60, 100%:79). We found that that 92% of biomarkers are significantly associated (Bonferroni-corrected p-value < 0.05) with age (Figure 2 and Table 3).

**Table 1:** Demographics of the dataset: sample sizes.

	All	non-Hispanic Whites	Hispanics	non-Hispanic Blacks	Others
All	27508 (100.0%)	12665 (46.0%)	7649 (27.8%)	5509 (20.0%)	1685 (6.1%)
Males	13443 (48.9%)	6273 (22.8%)	3649 (13.3%)	2692 (9.8%)	829 (3.0%)
Females	14065 (51.1%)	6392 (23.2%)	4000 (14.5%)	2817 (10.2%)	856 (3.1%)

**Table 2:** Demographics of the dataset: age distribution.

	All	non-Hispanic Whites	Hispanics	non-Hispanic Blacks	Others
All	46.3 (32-60)	47.9 (34-62)	44.7 (31-59)	46.0 (32-60)	43.4 (30-55)
Males	47.0 (33-61)	48.6 (35-62)	45.1 (31-60)	46.6 (33-61)	43.7 (30-56)
Females	45.7 (31-60)	47.1 (33-61)	44.3 (30-59)	45.5 (31-59)	43.1 (30-55)

The first number is the mean age of the demographic subgroup, the two number between parentheses are the 25th and 75th percentile of the age distribution.

**Table 3:** Biomarkers list: significance of the changes in biomarkers with age.

Biomarkers	Coefficients (sd/sd year)	neg-log-corrected-p-values	Pearson correlations
Upper Leg Length	-3.70	308	-0.245
Albumin	-2.60	175.9	-0.170
Lymphocyte number	-2.10	119.8	-0.141
Red blood cell count	-1.90	98.0	-0.128
60 sec. pulse	-1.90	95.5	-0.126
Total protein	-1.90	94.9	-0.126
Standing Height	-1.50	55.5	-0.097
Platelet count	-1.40	50.9	-0.093
Phosphorus	-1.10	30.8	-0.072
Lymphocyte percent	-1.00	27.9	-0.069
Chloride	-9.30x10^-1^	22.3	-0.062
Segmented neutrophils number	-9.00x10^-1^	20.8	-0.060
Mean platelet volume	-6.60x10^-1^	10.7	-0.044
Hematocrit	-6.20x10^-1^	9.4	-0.041
MCHC	-4.10x10^-1^	3.6	-0.027
Iron, refrigerated	-3.60x10^-1^	2.4	-0.024
Total calcium	-2.50x10^-2^	-1.6	-0.002
Monocyte number	3.60x10^-2^	-1.5	0.002
Segmented neutrophils percent	2.70x10^-1^	0.8	0.018
Total bilirubin	2.90x10^-1^	1.2	0.019
Basophils number	3.30x10^-1^	1.8	0.022
Alanine aminotransferase ALT	3.60x10^-1^	2.3	0.024
Albumin, urine	3.70x10^-1^	2.7	0.025
Eosinophils number	5.60x10^-1^	7.3	0.037
Basophils percent	6.60x10^-1^	10.7	0.044
Upper Arm Length	7.10x10^-1^	12.5	0.047
Arm Circumference	8.00x10^-1^	16.1	0.053
Sodium	9.70x10^-1^	24.0	0.064
Weight	1.00	25.7	0.066
Eosinophils percent	1.30	44.3	0.086
Monocyte percent	1.40	49.5	0.091
Aspartate aminotransferase AST	1.40	52.2	0.094
Alkaline phosphotase	1.60	63.3	0.103
Diastolic Blood pres	1.90	90.5	0.123
Potassium	1.90	97.5	0.127
Mean cell hemoglobin	2.00	100.6	0.129
Body Mass Index	2.00	106.2	0.133
Gamma glutamyl transferase	2.10	116.4	0.139
Creatinine	2.40	152.1	0.159
Bicarbonate	2.60	174.7	0.170
Triglycerides	2.70	187.9	0.176
Red cell distribution width	2.70	195.4	0.179
Lactate dehydrogenase LDH	3.00	248.0	0.202
Cholesterol	3.30	288.8	0.217
Osmolality	3.70	308	0.244
Waist Circumference	3.70	308	0.246
Glucose, serum	4.30	308	0.287
Blood urea nitrogen	4.90	308	0.325
Glycohemoglobin	5.30	308	0.353
Systolic Blood pres	6.40	308	0.423

The “Coefficients” column reports the coefficient obtained by performing a weighted regression on the biomarker against age. The “neg-log-corrected-p-values” column reports the corresponding p-values, Bonferroni corrected for the 50 tests. The “Pearson correlations” column reports the weighted correlation between age and the biomarker.

### Age-dependent correlations between biomarkers reveal structure changes with age

**Figure 3:**
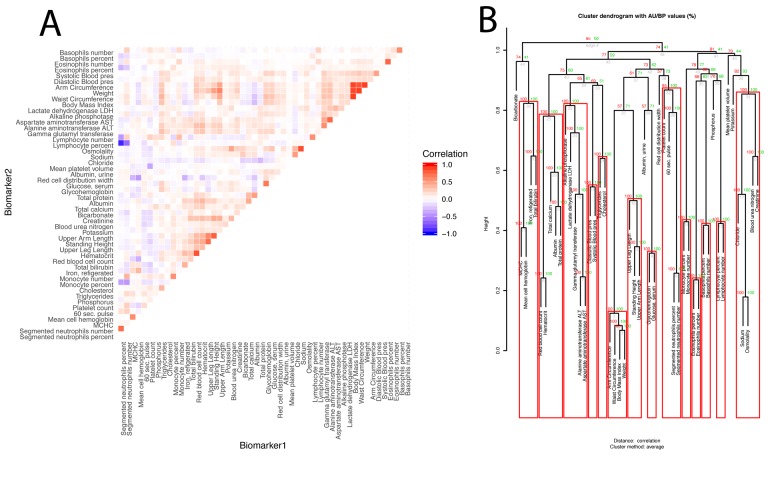
**Baseline correlation structure of the biomarkers. **(**A**) Correlation matrix of the biomarkers.****The biomarkers are ordered based on their clustering. Red is associated with a positive correlation, blue with a negative correlation. (**B**) Hierarchical clustering of the biomarkers. For each cluster, the number in green is the bootstrap probability (BP)--the percentage of bootstraps in which the cluster was present. The number in red is called the approximated-unbiased p-value (AU). AU is a better estimation of the unbiased p-value than BP, and the red boxes circle the significant clusters, based on this criterion, with alpha=0.95 (a cluster is marked as significant if its AU is greater than 95). The number in grey is the rank of the cluster, low numbers means the clustering happened early in the process. The height is the measure of the proximity between the two clusters being merged. The height is one minus the mean correlation between the two clusters, so two perfectly correlated biomarkers/clusters cluster at height zero, and two perfectly uncorrelated biomarkers/clusters cluster at height one. The first column compares males and females, the second column compares non-Hispanic Whites and Hispanics, the third column compares non-Hispanic Whites and non-Hispanic Blacks, and the fourth column compares the two control groups.

Next, we estimated the overall correlation structure of the biomarkers ((Figure 3AB). The mean of the absolute values of the correlations is modest (0.028; SE: 0.11).

**Figure 4:**
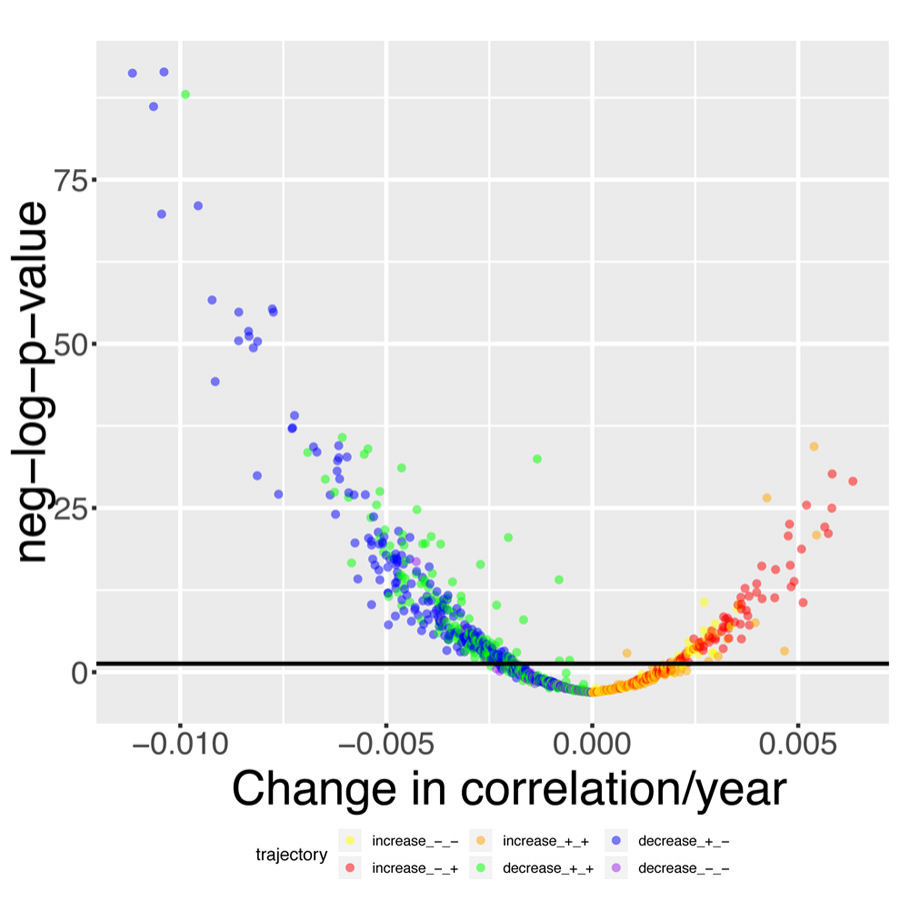
**Volcano plot of correlations changes with age.** The black horizontal line corresponds to the Bonferroni-corrected significance threshold (0.05). The green dots are correlations that decrease with age but remain positive. The blue dots are correlations that decrease with age, starting positive and ending negative. The purple dots are correlations that decrease with age and remain negative. The yellow plots are correlations that increase with age but remain negative. The red dots are correlations that increase with age, starting negative and ending positive. The orange dots are correlations that increase with age and remain positive.

**Figure 5:**
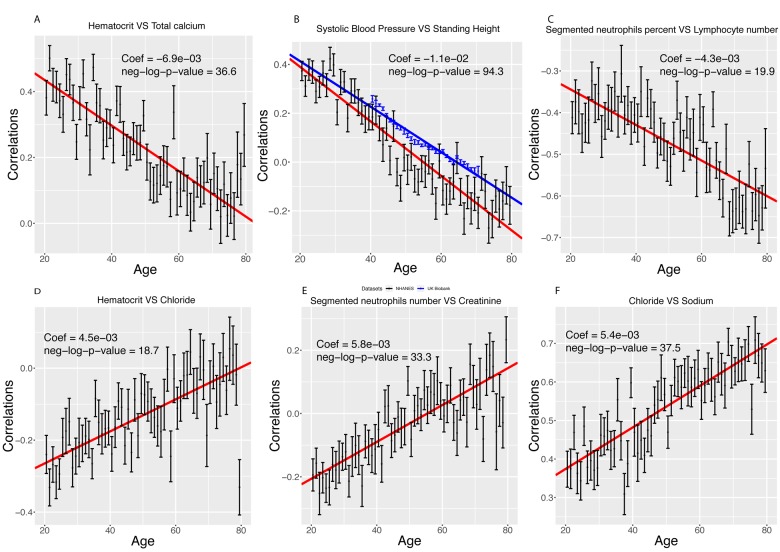
**Examples of different trajectories for changes in correlations with age. **(**A**) Hematocrit vs. total calcium. Example of a correlation that decreases with age but remains positive. (**B**) Standing height vs. systolic blood pressure. Example of a correlation that decreases with age, starts positive and ends negative. In red, we replicated the analysis on the UK Biobank cohort. (**C**) Percentage of segmented neutrophils vs. number of lymphocytes**.** Example of a correlation that decreases with age and remains negative. (**D**) Hematocrit vs. chloride. Example of a correlation that increases with age but remains negative. (**E**) Percentage of segmented neutrophils vs. creatinine. Example of a correlation that increases with age, starts negative and ends positive. (**F**) Chloride vs. sodium. Example of a correlation that increases with age and remains positive.

We found that 419 out of the 1,225 (33.2%) pairwise correlations show significant (Bonferroni-corrected p-value < 0.05) change with age after correcting for multiple testing (Figure 4). We classified these correlations with 6 different and general trends, as illustrated in Figures 4 and 5. 316 (25.8%) correlations significantly decreased with age (Figure 5A-C). They (1) start positive among young individuals and end negative among old individuals (189 findings [15.4%], such as standing height vs. systolic blood pressure, Figure 5A). Second, they (2) start positive and remain positive (123 findings [10.0%], such as hematocrit vs. total calcium, Figure 5B), Third, they (3) start negative and remain negative (four findings [0.3%], such as percentage of segmented neutrophils vs. number of lymphocytes, Figure 5C).

Inversely, we found 103 correlations (8.4%) that increase with age (Figure 5D-F). They (1) start negative and end up positive (65 findings [5.3%], such as percentage of segmented neutrophils vs. creatinine, Figure 5D), (2) start negative and remain negative (22 findings [1.8%], such as hematocrit vs. chloride, Figure 5E), or (3) start positive and remain positive (16 findings [1.3%], such as chloride vs. sodium, Figure 5F). A summary table of the different age trajectories can be found in Table 4.

**Table 4:** Distribution of the different types of trajectories for the changes in correlations with age.

	Increases	Decreases	Total
Switches sign	65 (5.3%)	189 (15.4%)	254 (20.7%)
Remains positive	16 (1.3%)	123 (10.0%)	139 (11.3%)
Remains negative	22 (1.8%)	4 (0.3%)	26 (2.1%)
Total	103 (8.4%)	316 (25.8%)	419 (33.2%)

We successfully replicated our top finding (standing height vs. systolic blood pressure) using 470,895 samples from the UK Biobank cohort [14] (Figure 5B). The correlation decreases by 0.011 unit/year in the NHANES participants (0.39 at age 20, -0.28 at age 80), and by 0.009 unit/year on the UK Biobank dataset (0.23 at age 40, -0.06 at age 71).

**Figure 6:**
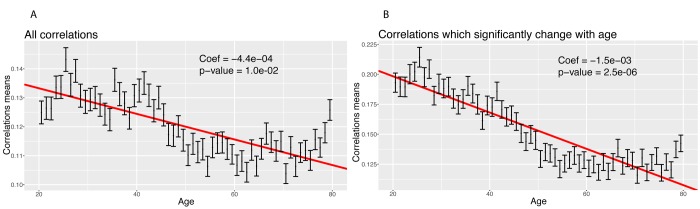
**Correlations: Change of the means of the absolute values of the correlations with age.** (**A**) Change of the mean of the absolute value of all the correlations with age. (**B**) Change of the mean of the absolute value of the correlations with age, limiting the analysis to correlations which significantly change with age.

We then tested for a global change in correlation amongst all 1,225 correlations. We tested for a change in the mean of the absolute values of the correlations with age (Figure 6). We confirmed a significant decrease in the means of the correlations with age, both when considering all the biomarkers (coefficient=-4.4x10^-4^ unit/year, p-value=2.4x10^-20^, Figure 6A), and those correlations that significantly changed with age only (coefficient=-1.5x10^-3^ unit/year, p-value=2.6x10^-56^, Figure 6B). Interestingly, we observed a non-linear trend. The mean absolute value of the correlations seems to decrease until the age of 50 and remain relatively stable after age 50. This suggests a non-linearity in the rate of aging.

**Figure 7:**
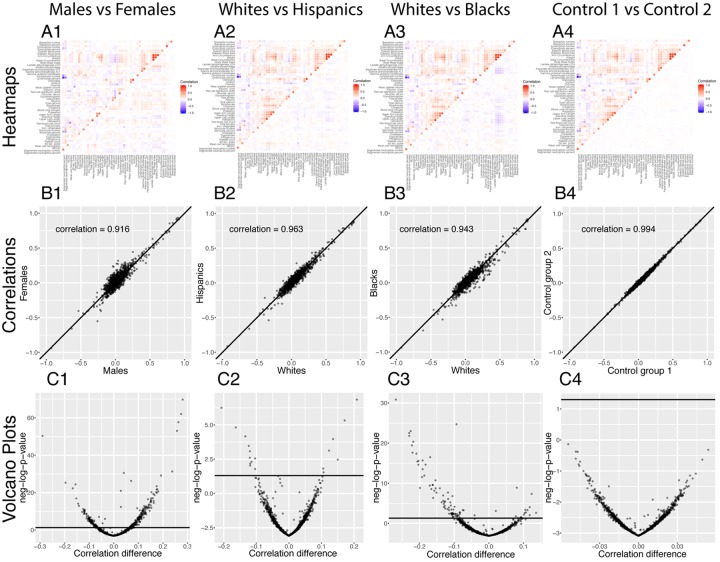
**Correlations: Baseline differences between demographics.** (**A**) Heatmap visualization of correlation structure. The upper left triangle shows the correlation structure for the first group of the comparison (e.g. males) while the lower right triangle of the matrix shows the difference between the second group and the first group of the comparison (e.g. difference between females and males). (**B**) Correlation between the 1,225 correlations of the first group (e.g. males) and the 1,225 correlations of the second group (e.g. females). The diagonal black line represents a perfect correlation. The further away from this line the points lie, the bigger the difference between the two groups, and the lower the correlation coefficient. (**C**) Volcano plots of the 1,225 differences in correlations between the groups. The horizontal black lines represent the threshold of significance of 0.05 for the Bonferroni corrected p‐values. The vertical axis is not shared between the plots. The first column compares males and females, the second column compares non-Hispanic Whites and Hispanics, the third column compares non-Hispanic Whites and non‐Hispanic Blacks, and the fourth column compares the two control groups.

We executed our pipeline on different sexes and ethnicities to compare the age-dependent correlation architecture between these groups (e.g., males versus females). We first established a baseline by comparing the correlation structure of the different demographics on the full age range (Figure 7).

296 (24.2%) correlations are significantly different between males and females. 190 (64% of 296) are higher for females than males. For example, the correlation between alkaline phosphatase and waist circumference is 0.32 for females versus 0.04 for males (p-value= 1.6x10^-70^), and the correlation between mean cell hemoglobin and red blood cell count is -0.40 for females and -0.54 for males (p-value=2.5x10^-13^). In contrast, 106 correlations (36% of 296) are higher for males than females. For example, the correlation between mean cell hemoglobin and red cell distribution width is -0.26 for males and -0.55 for females (p-value=5.0x10^-51^), and the correlation between standing height and weight is 0.44 for males and 0.28 females (p-value=4.0x10^-25^).

We also detected differences between ethnicities (non-Hispanic Whites versus Hispanics: 58 findings, 25 larger for Hispanics, 33 larger for non-Hispanic Whites, Figure 7 columns 2. non-Hispanic Whites versus non-Hispanic Blacks: 113 findings, 26 larger for non-Hispanic Blacks, 87 larger for non-Hispanic Whites, Figure 7 column 3.). Those differences were stronger than the differences between the controls (2 spurious findings, Figure 7 column 4), which further confirms their significance.

**Figure 8:**
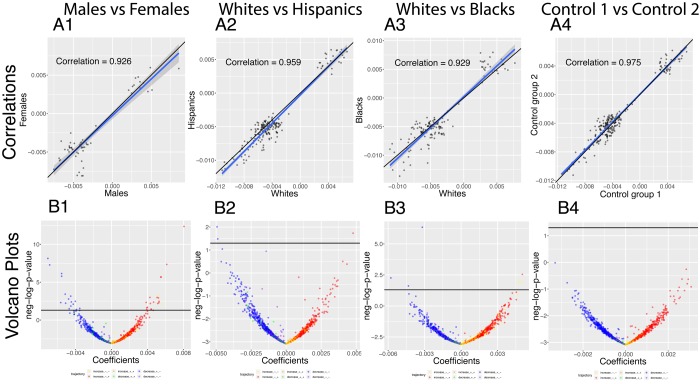
**Correlations: Differences in age trajectories between demographics.** (**A**) Correlations between the rates at which the correlations are changing in the two compared groups, only taking into account the correlations for which significant changes were detected in both compared groups (e.g. males and females). (**B**) Volcano plots showing differences in the rates of change of correlations with age between different demographic groups. The horizontal black lines represent the threshold of significance of 0.05 for the Bonferroni corrected p-values.

We analyzed which correlations trend significantly differently with age in different demographics (Figure 8). We detected 36 significant sex differences in the age trajectories of correlations (Figure 8B column 1). 20 of them show an increase with age (1.6%). For example, cholesterol vs. albumin has a difference in correlation trajectory (p-value=4.0x10^-13^). In females it increases from -0.22 at age 20 to 0.31 at age 80; in contrast, males exhibit no significant change in correlation (0.12 on average). 16 of the correlations’ differences showed a decrease with age (1.3%). For example, blood urea nitrogen vs. albumin has a difference in correlation trajectory (p-value=7.9x10^-9^). In females it decreases from 0.38 at age 20 to -0.15 at age 80; in contrast, males exhibit no significant change in correlation (0.06 on average).

We detected few differences in age trajectories of correlations between ethnicities. Specifically, we detected three differences in correlations’ trajectories between non-Hispanic Whites and Hispanics (Figure 8 columns 2) and eight differences between non-Hispanic Whites and non-Hispanic Blacks (Figure 8 columns 3).

The comparison between the two control groups, on the other hand, did not exhibit any differences. This suggests that the findings for the comparisons between the sexes and the ethnic groups are not spurious. A summary table of the trajectories of the correlations in different demographic groups can be found in Table 5.

**Table 5:** Distribution of the age trajectories of the correlations, the predictabilities and the regression coefficients in different demographics.

	Trajectory	All	Males	Females	Whites	Hispanics	Blacks	Control 1	Control 2
Correlations (out of 1225)	Decrease ++	123 (10.0%)	28 (2.29%)	30 (2.45%)	101 (8.24%)	47 (3.84%)	34 (2.78%)	85 (6.94%)	74 (6.04%)
	Decrease -+	189 (15.4%)	57 (4.65%)	72 (5.88%)	157 (12.8%)	143 (11.7%)	104 (8.49%)	147 (12.0%)	139 (11.3%)
	Decrease --	4 (0.33%)	3 (0.24%)	3 (0.24%)	1 (0.08%)	2 (0.16%)	1 (0.08%)	4 (0.33%)	4 (0.33%)
	Increase --	22 (1.80%)	9 (0.73%)	11 (0.90%)	6 (0.49%)	3 (0.24%)	5 (0.41%)	5 (0.41%)	5 (0.41%)
	Increase -+	65 (5.31%)	19 (1.55%)	45 (3.67%)	56 (4.57%)	17 (1.39%)	35 (2.86%)	51 (4.16%)	45 (3.67%)
	Increase ++	16 (1.31%)	15 (1.22%)	13 (1.06%)	14 (1.14%)	7 (0.57%)	5 (0.41%)	14 (1.14%)	7 (0.57%)
Predictabilities (out of 50)	Decrease	17 (34%)	13 (26%)	8 (16%)	6 (12%)	9 (18%)	2 (4%)	1 (2%)	5 (10%)
	Increase	5 (10%)	3 (6%)	4 (8%)	3 (6%)	2 (4%)	2 (4%)	3 (6%)	3 (6%)
Regression coefficients (out of 2450)	Decrease +-	13 (0.53%)	12 (0.49%)	13 (0.53%)	8 (0.33%)	15 (0.61%)	12 (0.49%)	12 (0.49%)	8 (0.33%)
	Decrease --	3 (0.12%)	1 (0.04%)	2 (0.08%)	2 (0.08%)	4 (0.16%)	1 (0.04%)	2 (0.08%)	1 (0.04%)
	Decrease ++	4 (0.16%)	2 (0.08%)	4 (0.16%)	2 (0.08%)	0 (0.00%)	1 (0.04%)	3 (0.12%)	2 (0.08%)
	Increase -+	7 (0.29%)	6 (0.24%)	8 (0.33%)	3 (0.12%)	10 (0.41%)	7 (0.29%)	8 (0.33%)	8 (0.33%)
	Increase --	2 (0.08%)	3 (0.12%)	3 (0.12%)	0 (0.00%)	1 (0.04%)	1 (0.04%)	0 (0.00%)	0 (0.00%)
	Increase ++	11 (0.45%)	12 (0.49%)	6 (0.24%)	8 (0.33%)	3 (0.12%)	5 (0.20%)	8 (0.33)	5 (0.20%)

Finally, we detected a difference between sexes in the rate at which the mean of the absolute values of the correlations that significantly change with age decreases. It decreases 37% faster for males (from 0.214 at age 20 to 0.165 at age 80) than for females (from 0.198 at age 20 to 0.162 at age 80).

### Predictability of biomarkers is strongly age dependent

**Figure 9:**
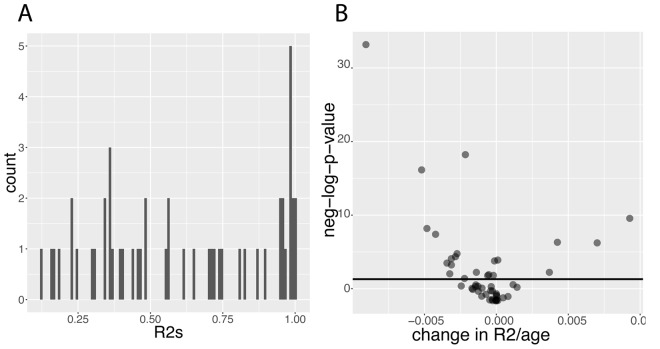
**Predictabilities: Baseline predictabilities for the 50 biomarkers and significance of their changes with age. **(**A**) Histogram of the predictabilities.****Histogram of the predictabilities (R^2^) obtained on the 50 biomarkers using the full 27,508 samples. (**B**) Volcano plot of the changes in predictabilities with age.****The horizontal black lines represent the threshold of significance of 0.05 for the Bonferroni corrected p-values.

Next, we sought to estimate how predictions of biomarkers change with age. We hypothesized that first, biomarkers are predictive of other biomarkers, and second, that these predictions significantly change with age. We established baseline results using the full cohort (Figure 9A) by predicting each of the biomarkers using all the other biomarkers. The mean R^2^ was 0.618, the standard deviation 0.294, the minimum 0.120 and the maximum 0.999.

**Figure 10:**
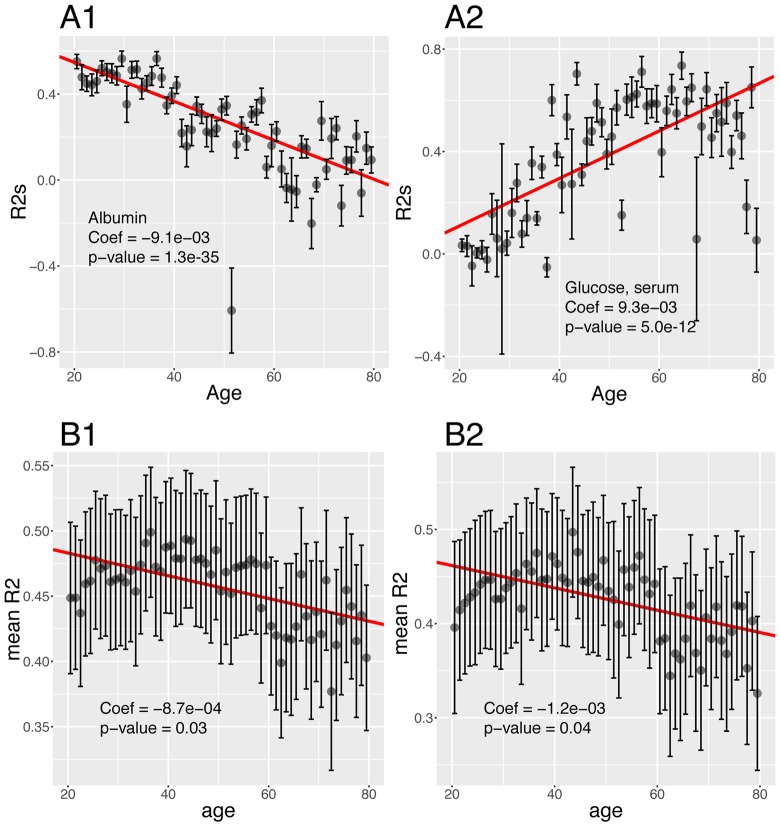
**Changes of the predictabilities (R^2^) with age. **(**A**) Predictabilities: Examples of different age trajectories. (**A1**) Albumin. Example of a biomarker whose predictability decreases with age. (**A2**) Glucose, serum. Example of a biomarker whose predictability increases with age. (**B**) Change of the means of the predictabilities with age. (**B1**) Change of the mean of all the predictabilities with age. (**B2**) Change of the mean of the predictabilities with age, limiting the analysis to biomarkers whose predictability significantly change with age.

Then, we tested for changes with age and found that the prediction accuracy of 22 (44%) of the biomarkers significantly change with age (Figure 9B). For example, we found that 55% of the variance of albumin can be explained by other biomarkers in people at age 20, whereas 0.00% of the variance can be explained when people are 80 (Figure 10A1). Inversely, we found that only 11% of the variance of serum glucose levels can be explained by other biomarkers at age 20, whereas 67% of it can be explained at age 80 (Figure 10A2). We found that the average predictability decreases by 8.7x10^-4^ unit/year over all the biomarkers year over year (p-value=0.03) (Figure 10B1).

**Figure 11:**
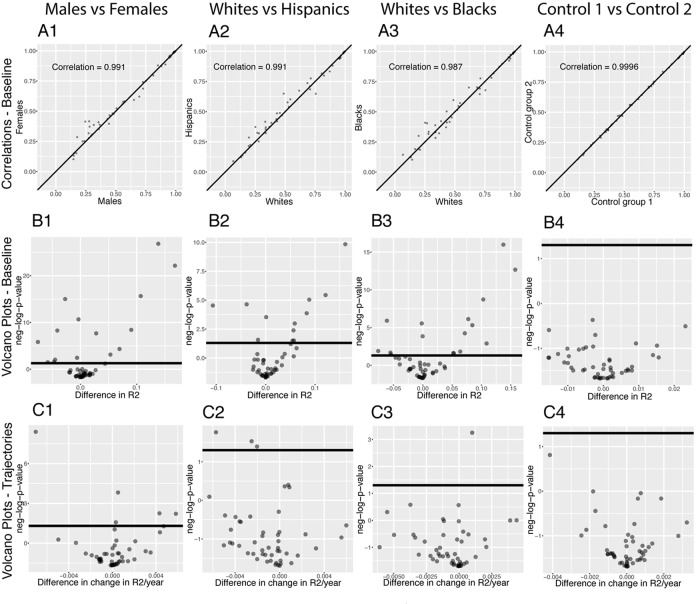
**Predictabilities: Differences between demographics.** (**A**) Correlation between the predictabilities of the 50 biomarkers in the first group and the predictabilities of the 50 biomarkers in the second group. The diagonal black line represents a perfect correlation. The further away from this line the points lie, the bigger the difference between the two groups, and the lower the correlation. (**B**) Volcano plots reporting the significance and the size of the baseline (calculated on the full age range 20-80) differences between the groups. The horizontal black lines represent the threshold of significance of 0.05 for the Bonferroni corrected p-values. (**C**) Volcano plots showing which predictabilities are changing at significantly different rates with age in different demographic groups. The horizontal black lines represent the threshold of significance of 0.05 for the Bonferroni corrected p-values.

We investigated the difference in predictabilities between sexes and ethnicities by running our pipeline on different demographic groups. First, we established baseline differences, looking at the full age range (Figure 11A and 11B). We detected 15 differences between sexes. 8 biomarkers show higher predictability in males versus females, such as alanine aminotransferase ALT (R^2^=0.70 for males, R^2^=0.62 for females, p-value=1.5x10^-6^). In contrast, 7 biomarkers are better predicted in females than males, such as red cell distribution width (R^2^=0.25 for males, R^2^=0.41 for females, p-value=7.2x10^-23^).

We detected differences in predictabilities between ethnicities, as well. 3 show higher predictability in non-Hispanic Whites versus Hispanics, such as blood urea nitrogen (R^2^=0.76 for non-Hispanic Whites, R^2^=0.65 for Hispanics, p-value=3.2x10^-5^), and 9 are better predicted for Hispanics, such as glycohemoglobin (R^2^=0.49 for non-Hispanic Whites, R^2^=0.65 for Hispanics, p-value 1.6x10^-10^). 6 were better predicted for non-Hispanic Whites than for non-Hispanic Blacks, such as Blood urea nitrogen (R^2^=0.76 for non-Hispanic Whites, R^2^=0.68 for non-Hispanic Blacks, p-value=0.01), and 10 were better predicted for non-Hispanic Blacks, such as red cell distribution width (R^2^=0.25 for non-Hispanic Whites, R^2^=0.41 for non-Hispanic Blacks, p-value=2.0x10^-13^). There were no significant findings detected when comparing the controls, confirming the signal of findings above.

Next, we tested for significant differences between demographic groups in the trajectories of predictabilities with age (Figure 11C). We detected different age trajectories of the predictabilities between males versus females. Four of them (10%) showed an increase of the signed difference between the R^2^s values with age, such as alanine aminotransferase. For males, the R^2^ value decreases from 0.65 to 0.45 whereas for females, there was no significant change in R^2^ value (0.47 on average). In contrast, in females, albumin has an R^2^ value of 0.55 at age 20 and 0.05 at age 80; however, in males, the R^2^ value does not significantly change (0.22 on average). We detected fewer differences between ethnicities (three differences between non-Hispanic Whites and Hispanics, and one difference between non-Hispanic Whites and non-Hispanic Blacks).

### Features that drive prediction of biomarkers change with age

**Figure 12:**
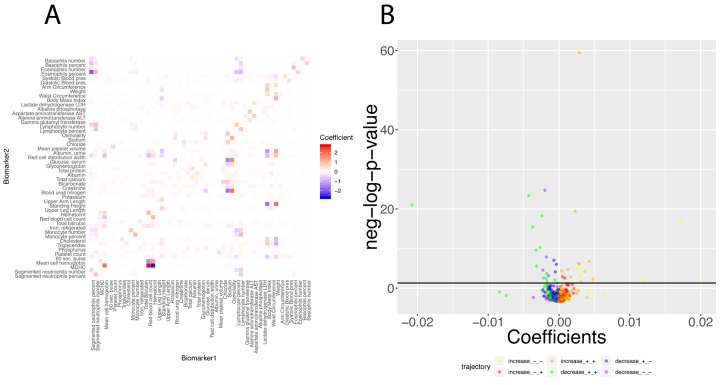
**Regression coefficients: Baseline regression coefficients for the prediction of the 50 biomarkers and significance of their changes with age. **(**A**) Heatmap of the regression coefficients on the full cohort. Each column corresponds to the 49 coefficients used to predict the values for one of the 50 biomarkers. (**B**) Volcano plot of the changes of the regression coefficients with age.The black horizontal line corresponds to the 0.05 significant threshold, after Bonferroni correction. The green dots are coefficients that decrease with age but remain positive. The blue dots are coefficients that decrease with age, starting positive and ending negative. The purple dots are coefficients that decrease with age and remain negative. The yellow plots are coefficients that increase with age but remain negative. The red dots are coefficients that increase with age, starting negative and ending positive. The orange dots are coefficients that increase with age and remain positive.

**Figure 13:**
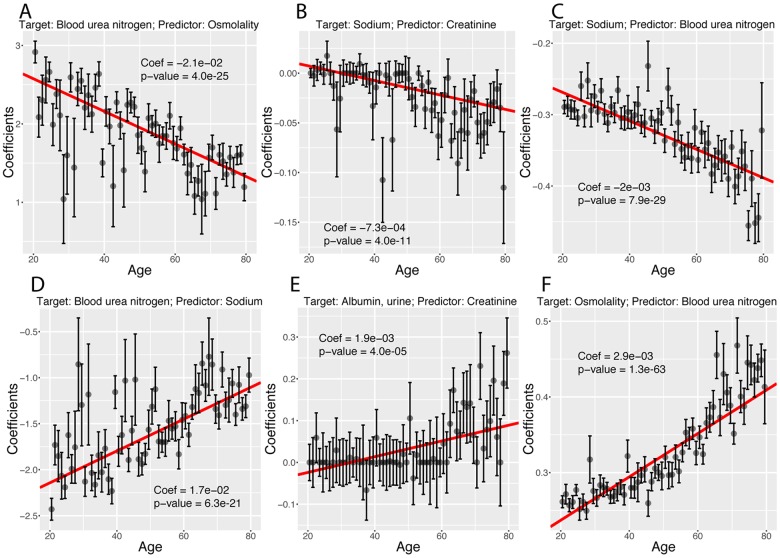
**Examples of different trajectories for changes in regression coefficients with age. **(**A**) Target: Blood urea nitrogen. Predictor: Osmolality. Example of a coefficient that decreases with age but remains positive. (**B**) Target: Sodium. Predictor: Creatinine. Example of a coefficient that decreases with age, starts positive and ends negative. (**C**)****Target: Sodium. Predictor: Blood urea nitrogen. Example of a coefficient that decreases with age and remains negative. (**D**) Target: Blood urea nitrogen. Predictor: Sodium. Example of a coefficient that increases with age but remains negative. (**E**) Target: Albumin, urine. Predictor: Creatinine. Example of a coefficient that increases with age, starts negative and ends positive. (**F**) Target: Osmolality. Predictor: Blood urea nitrogen. Example of a coefficient that increases with age and remains positive.

Finally, we investigated what exact biomarker variables influence the predictions for every age. We first estimated these coefficients on the whole cohort (Figure 12A), then examined what coefficients change with age (Figures 12B, 13). We found that only 40 out of the 2,450 (1.6%) coefficients significantly change with age. 20 (0.8%) increase and 20 (0.8%) decrease. For example, osmolality and sodium become weaker predictors of blood urea nitrogen with age (Figure 13A and 13D), while blood urea nitrogen becomes a stronger predictor of both osmolality and sodium with age (Figure 13C and 13F). We also observed that predictor selection is affected by age. For example, sodium and urine albumin are not selected as predictors of creatinine for young people but become good enough predictors to be selected at older age (Figure 13B and 13E).

**Figure 14:**
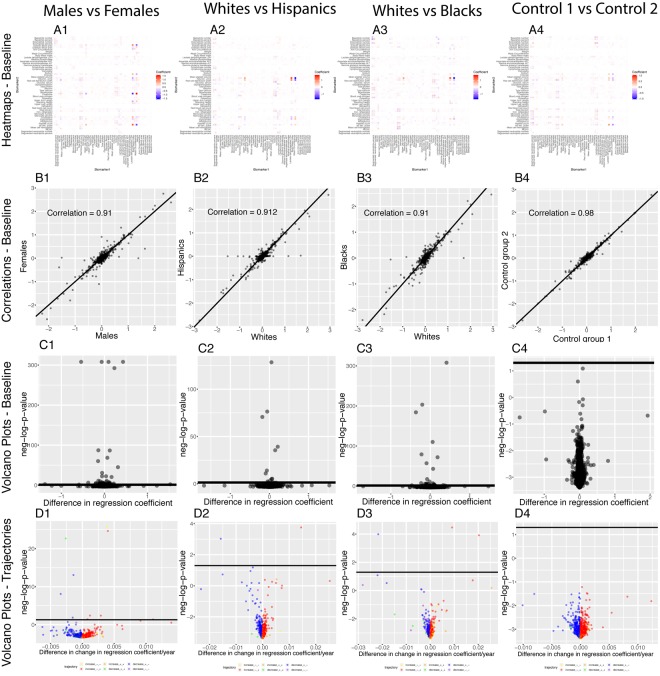
**Regression coefficients: Differences between demographics.** (**A**) Heatmaps displaying the baseline differences in the regression coefficients between demographic groups. (**B**) Correlation between the 2,450 regression coefficients in the first group and the 2,450 regression coefficients in the second group. The diagonal black line represents a perfect correlation. The further away from this line the points lie, the bigger the difference between the two groups is, and the lower the correlation coefficient is. (**C**) Volcano plots reporting the baseline (calculated on the full age range 20-80) differences in regression coefficients between the groups. The horizontal black lines represent the threshold of significance of 0.05 for the Bonferroni corrected p-values. (**D**) Volcano plots showing which regression coefficients are changing at significantly different rates with age in different demographic groups. The horizontal black lines represent the threshold of significance of 0.05 for the Bonferroni corrected p-values.

We found 70 (2.9%) significant baseline differences between males and females, 16 (0.65%) between non-Hispanic Whites and Hispanics, 29 (1.18%) between non-Hispanic Whites and non-Hispanic Blacks) and 1 (0.04%) false discovery between the two controls. We found even fewer differences for the age trajectories: 10 (0.4%) differences between sexes, 2 (0.08%) differences between non-Hispanic Whites and Hispanics, 3 (0.012%) differences between non-Hispanic Whites and non-Hispanic Blacks and 0 (0.00%) differences the controls (Figure 14).

### Summary

Table 6 summarizes which percentage of correlations, predictabilities and regression coefficients are significantly different from zero at baseline. It also reports which percentage of correlations, predictabilities and regression coefficients significantly change with age. Finally, it recapitulates the percentage of correlations, predictabilities and regression coefficients that are different between sexes and between ethnicities, both at baseline and in term of changes with age.

**Table 6:** Summary results: number and percentage of findings in terms of correlations, predictabilities and regression coefficients in the different demographic groups.

Analysis		**All**	**Males vs Females**	**Whites vs Hispanics**	**Whites vs Blacks**	**Control 1 vs Control 2**
**Correlations** (out of 1225)	Baseline	883 (72.1%)	296 (24.2%)	18 (1.47%)	94 (7.67%)	0 (0.00%)
	Trajectories	419 (34.2%)	36 (2.94%)	3 (0.24%)	6 (0.49%)	0 (0.00%)
**Prediction accuracies** (out of 50)	Baseline	50 (100.0%)	15 (30.0%)	12 (24.0%)	16 (32.0%)	0 (0.0%)
	Trajectories	22 (44.0%)	5 (10.0%)	3 (6.0%)	1 (2.0%)	0 (0.0%)
**Regression coefficients** (out of 2450)	Baseline	625 (25.5%)	70 (2.86%)	16 (0.65%)	29 (1.18%)	1 (0.04%)
	Trajectories	40 (1.63%)	10 (0.41%)	2 (0.08%)	3 (0.12%)	0 (0.00%)

The “all” column reports the number and percentage of findings that were significantly (Bonferroni corrected p-value < 0.05) different from zero during the analysis of the whole dataset, demographically wise. For example, 883 (72.1%) is the number of baseline correlations that are significantly different from zero, including the samples of all sexes and ethnicities in the analysis. The remaining columns are the number and percentage of significant differences detected between the two groups mentioned in the column name. For example, 296 (24.2%) is the number of correlations which are significantly different between males and females, when including the samples from the full age range (20-80 years). The “baseline” rows report the number and percentage of positive findings (values significantly different from 0) calculated on the full age range. The “trajectories” rows report the number and percentage of positive findings (value significantly different from 0) for the rates of changes with age.

We also share our results on an interactive Shiny app: http://apps.chiragjpgroup.org/Aging_Biomarkers_Co-Dependencies/.

## DISCUSSION

In summary, we have shown that the co-dependency structure of biomarkers changes as humans age. Further, we have evidence to support that the structure is different in males and females and between ethnicities. In general, differences between sexes are stronger than differences between ethnicities, and more differences were found between non-Hispanic Whites and non-Hispanic Blacks than between non-Hispanic Whites and Hispanics.

We found that 92% of the biomarkers, 33% of the pairwise correlations, 44% of the predictabilities and 1.6% of the regression coefficients show significant change with age.

Demographics played an influential role. First, we found significant differences between sexes at baseline (44% of the correlations, 30% of the predictabilities and 2.9% of the regression coefficients). In term of age trajectories, we found that sex influences 2.7% of the correlations, 10% of the predictabilities and 0.4% of the regression coefficients. Last, we found fewer baseline and trajectories differences between non-Hispanic Whites and non-Hispanic Blacks, and even fewer differences between non-Hispanic Whites and Hispanics (Table 6).

Some of our results may be prone to misinterpretation due to generational bias. For example, the physiological indicator that was the most negatively correlated with age was upper leg length. This can be explained by the fact that people who are currently old are shorter than they would have been if they had had access to contemporary diets and quality of life of later generations. People who are old in 2018 have shorter upper leg length than young people in 2018, but they do not have shorter upper leg than in their youth. Using a longitudinal cohort, we would therefore not observe this generational bias.

Our strongest finding regarding changes in correlations with age was the correlation between height and systolic blood pressure. Others have reported similar findings. First, Bourgeois et al. [15] reported a negative association between height and blood pressure using a using representative cohort from the 1999-2006 NHANES dataset, but the apparent trend reversed in young people. Fujita et al. [16] found a positive correlation between height and blood pressure for Japanese children. Lu et al. [17] found that the ratio between modified blood pressure and height decreases with age in Han children. Interestingly, the correlation decreases from 0.39 for young people to -0.28 for old people, but it only decreases from 0.09 to -0.13 in males, and from 0.07 to -0.20 in females. A potential explanation is that on average males remain taller than their female counterparts in every age group; however, young males have on average higher blood pressure than young females, whereas older males have on average lower blood pressure than their female counterparts [18].

A generational bias could explain the slight shift observed when replicating our top correlation finding (height vs. systolic blood pressure), since the UK Biobank participants were recruited after the NHANES participants. Another potential explanation may be a geographical or trans-continental UK/USA environmental effect.

We hypothesize that the shrinkage of the relationship between biomarkers may be indicative of the aging process. Specifically, we found that, overall, the correlation between biomarkers and their predictability significantly decreases with age. These findings could be explained by a progressive loss of the homeostasis, or by the accumulation of the effects of the environment as we age. Both those effects have the potential to decorrelate initially correlated variables. For example, we found that albumin levels could not be predicted in old people; however, 55% of its variance can be explained in young people. Albumin levels are affected by both the diet and several common diseases, such as liver failure, heart failure, kidney damage or enteropathy [19]. Potentially cumulative effects with time influence the liver more than other organ systems as humans age.

A limitation that we faced in this study was the limited number of samples available to dissect the relationship between ethnicity and sex in some age ranges. To compare different demographic groups and different age bins, we used the minimum of those different sample sizes over the different age groups and demographics to train the models before generating the predictabilities. This decreased our power to detect changes with age in predictabilities. NHANES also is limited in the information regarding ethnicity beyond the groups studied in this investigating, which is seen in the absence of data from the Asian population. Last, the lack of repeated measures to estimate inter-person correlation was a limitation as well; and therefore, providing a personal level correlation was not possible. Because correlations are a group measure, we can follow the trends in groups but not in individuals.

It is striking that a large number of significant changes for the correlations translates into such a small number of changes in regression coefficients. One possible explanation is that the “regularization” analytic procedure that we used to select biomarker predictors constrained most of the small coefficients to zero. Another plausible explanation is that the values of the coefficients are, in fact, changing, but because there is a large confidence interval on the values of those coefficients, we are underpowered to detect significant changes (changes that are statistically non-zero).

Another limitation of our study is that the biomarkers selected are not typical chronological age biomarkers, such as telomere length or the emerging DNA methylation clock [7]. Rather, this project aimed to understand how the correlation structure of 50 human clinical biomarkers changes with age. Therefore, in the future, we would like to investigate the correlation structure of chronological age biomarkers, such as the ones presented in the MARK-AGE study [20] or the 44 biomarkers associated with frailty by Cardoso et al. [21] (e.g. telomeres length, DNA methylation, inflammatory miRNAs, interleukin 6, C-X-C motif chemokine ligand 10, C-X3-C motif chemokine ligand 1). Aside from aging biomarkers, it would be valuable to study the correlation structure of biomarkers focused on different organ systems. Our current panel is focused on blood biomarkers and anthropometrics.

The findings in this paper are relevant when building prediction algorithms, specifically those that utilize regularization, such as LASSO [22–28]. The biomarker variable selection process depends on the correlations between the predictors. If two predictors are heavily correlated, one of them will get discarded by the model. Because models often do not take into account time-dependency of biomarkers and are usually built on the entire cohort, the regularization process will only take into account the average correlation on the full age range covered by the data set. Based on the numerous positive findings in this paper with respect to changes with age, we advise to first investigate if the correlations between the biomarkers used change with age. If they do, a solution is to add interaction terms between age and the biomarkers to the model. This is not always done (e.g. [29]) as it increases the number of predictors and the computational cost. Our work suggests that this addition might improve the performance of the model. We offer a R/Shiny web application to interactively display and explore our results to help researchers navigate subsets of biomarkers in depth before building models.

If the generational effect component can be accounted for, calculating the rates at which correlations, predictabilities, or regression coefficients are changing in different populations could also be used to monitor and compare their aging rate. For example, we hypothesize that populations in poor socioeconomic conditions will show earlier and faster change in their correlation structure than populations of high socioeconomic status. We also hypothesize that the age trajectories differ according to smoking and dietary status. Comparisons between sexes and ethnicities are a possibility as well, but they rely on the assumption that the biology underlying aging is the same in the different groups, and that differences can be imputed to different aging rates. If one is willing to make this assumption, our finding that the mean absolute value of the correlations that significantly change with age is decreasing 37% faster in males than females suggests that males are aging faster than females, which could explain their shorter longevity [30–33].

As future work, the assessment of changes in the variance of the correlation could provide further insight about the aging process. We hypothesize that with age, the variance of the correlations will increase as people get diagnosed with chronic and age-related disease. We investigated preliminarily whether the variances of the correlations/R^2^s/coefficients change with age and differ between demographics; however, we were underpowered to detect any robust findings.

We observed several non-linear age trajectories, such as the decrease of the mean of the absolute values of the correlations or the change in predictability of serum glucose, which suggests non-linearities in the rate of aging. Testing for non-linearity of the trajectories of biomarkers, correlations, R^2^s and coefficients may also be valuable to investigate potential non-linearities in the rate of aging.

Finally, much work is being done to find reliable human aging biomarkers and build biological age predictors. Many predictors now combine several biomarkers. [6, 8]. The findings of this paper call for a chronological age-dependent definition of biological age.

## MATERIALS AND METHODS

### Dataset

We used the 1999-2014 cohorts of the National Health and Nutrition Examination Survey (NHANES) dataset (Figure 1A), a cross-sectional survey. We first selected the biomarkers under the categories “Laboratory” (blood biomarkers) or “Examination” (anthropometric biomarkers). We acknowledge the different definitions of biomarkers. For the scope of this paper, we refer to “biomarkers” when describing the variables selected, including phenotypes such as height. When a biomarker had its value recorded in both the American and the international metric system, we excluded the latter. For patients who had more than one measure for blood pressure, we took the average. We excluded from the analysis the individuals younger than 20 years old and the individuals aged 80 or above because of the limited sample size in those age ranges. We iteratively excluded the samples and biomarkers with the most missing values from the dataset until we were left with a complete matrix of 27,508 samples and 54 biomarkers, out of the initial 82,440 samples and 1,308 biomarkers (Figure 1B). Out of the initial 1,308 biomarkers, some correspond to repeated measures of a single biomarker, such as blood pressure. Some of those 1,308 biomarkers are measured in a large number of individuals, while some others were only measured on a subset of the cohort, hence the need to extract a complete matrix from this scarce starting matrix.

We calculated the pairwise correlations between those 54 biomarkers and filtered out four other biomarkers, which were more than 90% correlated with another (total cholesterol correlated with cholesterol, hemoglobin correlated with hematocrit, white blood cell count correlated with neutrophils number, mean cell volume correlated with mean cell hemoglobin). We performed logarithmic or exponential transformations on the biomarkers to make their distributions look more Gaussian then we centered and scaled the biomarkers using the survey weights. A description of the demographics can be found in Tables 1 and 2. We tested for a change of the biomarkers with age using a weighted linear regression (the significance threshold was 0.05, after Bonferroni correction.)

We replicated our top finding in term of changes in correlation with age using the UK Biobank dataset. After preprocessing the 502,628 samples for missing values, we obtained a sample size of 470,895. More details about the demographics of this cohort can be found in Tables S1 and S2.

### Calculation of the correlation structure of the biomarkers

### *Hierarchical clustering of the biomarkers*

We performed weighted hierarchical clustering using the survey weights to group correlated biomarkers. We corrected the clusters for age, sex and ethnicity using partial correlations. We bootstrapped the clustering 1,000 times to estimate the significance of all the different clusters with the R library “pvclust” [34]. We used the default value of 0.95 for alpha to determine the significance of each cluster. (The alpha parameter controls how frequently the cluster must be present in the bootstrapped trees to be considered significant.)

### *Calculation of the age trajectories of the correlations*

First, we split the dataset by age (Figure 1C). For individuals in each age (i.e. 20, 21, up to 79), we computed a weighted Pearson moment correlation between each of the 50 biomarkers in participants of the same age, a total of 1,225 correlations (Figure 1D). We bootstrapped the calculation of each correlation for each age 1,000 times to obtain a confidence interval for each correlation at a given age. For each correlation, we performed a “meta-regression” regressing the correlation with age to test for significant change with age (Figure 1E). Because we tested for significant changes with age in 1,225 pairwise correlations, we corrected the p-values obtained from the meta-regressions for multiple testing using the Bonferroni method (0.05 /1,225). If the p-value was so low that it was recorded as zero on the computer, we reported the negative log p-value to be superior to 308 (corresponding to the negative log p-value of the smallest number that could be coded on the computer).

### *Calculation of the age trajectories of the mean of the absolute values of the correlations*

We tested for a trend in the evolution of the 1,225 correlations. At each age, we calculated the mean and the standard deviation of the absolute values of the 1,225 correlations. We then performed a meta-analysis to test if the mean of the absolute values of the correlations was significantly changing with age.

We also performed a similar calculation, this time after excluding from the analysis the 806 correlations that we found do not significantly change with age.

### *Calculation of the baseline differences for the correlations between sexes and ethnicities*

For each correlation, we determined the baseline difference between demographic groups. For each demographic group, we computed the correlation using the full age range (20-80) and bootstrapped this calculation 1,000 times to obtain confidence intervals. We then calculated the p-values using the mean and the standard deviations of the two groups. Because we tested 1,225 pairs of biomarkers correlations, we Bonferroni corrected the p-values obtained accordingly. We used a threshold of 0.05 after correction for significance.

### *Calculation of the differences between demographic groups in the age trajectories of the correlations*

First, we split the data by demographic groups and by age. For each group and for each age, we calculated the 1,225 correlations and their standard deviation (using 1,000 folds bootstrapping). For each age and for each correlation, we then calculated the differences between the groups, as well as the variance of the difference (the sum of the variance in the two groups compared). For each group comparison and for each correlation, we performed a meta-regression using the values and the standard deviation of the difference in correlation between the two groups. We Bonferroni corrected the p-value obtained and used a threshold of 0.05/1,225 for significance.

### Calculation of the predictability for each biomarker

To evaluate the predictability for each biomarker, we first split the relevant cohort (in terms of demographics and age range) into a training and a testing set (50/50). We fit an elastic net with alpha=0.5 and we picked the optimal lambda among 100 values suggested by the glmnet R package using a 10 folds cross-validation on the training set. We then generated predictions for the testing set. Next, we switched the training and the testing set and used the same procedure to generate predictions on the other half of the dataset. We then merged the prediction of both halves and compared the predictions to the actual values over all the samples using a non-corrected coefficient of determination (R-squared) to report the predictability. We bootstrapped the calculation of the R-squared 1,000 times to obtain its standard error.

We observed that our models performed significantly better when trained on 600 samples than when trained on 200 samples. Because different age bins have different sample size, and that older age bins tend to have a smaller sample size, we would have obtained biased results that might have a decrease in the predictabilities of the biomarkers with age. We corrected for this bias by using the same number of samples for each age bin when comparing predictabilities for different ages. Similarly, we used the same number of samples to compare between sexes and between ethnicities. Taking the minimum of the sample sizes sometimes left us with too few samples to reach significance so, as a consequence, we used a larger age window for those analyses (Table 7). The windows did not overlap, and each age bin analysis is independent from the other others.

**Table 7:** Sliding window sizes and sample sizes used for the different demographics for the calculation of the changes in predictabilities with age.

Demographics	Window Size (years)	Sample size
All	1	180
Sexes	2	179
Ethnicities	5	164

We investigated the age trajectories of predictabilities in two ways. First, as described above, we built a model for each age bin, and used it to estimate the predictability on this same age range. Secondly, we built a single model on the full age range, and then evaluated how this model performed to predict the values of the biomarkers on different age bins. The advantage of the first model using a sliding window is that each model is specific to the age bin and can be analyzed in the context of the correlations and regression coefficients of the same age bin. The downside is the small sample size available to train the models. The advantage of the second model is the larger training sample size. Its downside is that it is not age bin specific and can therefore not be analyzed in the context of the age bin specific correlations and regression coefficients. To be concise, we only present the results obtained using a sliding window in this paper, but the results obtained using a single model can be found on the website.

We examined the demographic differences and the age dependence of predictabilities following the same pipeline as we did when we examined the correlations between biomarkers. We first established a baseline on the full cohort and age range before performing the analysis on the difference age bins and testing for a significant change of the mean. Then we looked at demographic differences both for the baseline and for the age trajectories.

For some analyses, we obtained negative R^2^ values. While it is unusual, this occurs when the performance of a model on the testing set is poorer than predicting the mean target of the testing set for each sample.

### Calculation of the regression coefficients for the prediction of each biomarker

To calculate the regression coefficient, we built a model using the same protocol described above to evaluate the predictabilities, but without splitting the data into a training and a testing set. To obtain the variance of the coefficients, we used 100-fold parametric bootstrapping. The pipeline we followed was identical to the one used to explore age and demographics changes for correlations and predictabilities.

### We performed the pipeline on “control groups”

In order to have a negative control for our findings with respect to sex and ethnicity differences, we built control groups. We randomly split the samples into two groups and performed the entire pipeline on those two groups like we did on each sex and ethnicity. By comparing the difference between those two groups, we estimated how much of the differences between sexes and ethnicities were due to random sampling, and how much were due to biological differences.

### Report of corrected p-values

P-values are probabilities and therefore take values between zero and one, while the negative log p-values have an upper limit of one. However, we chose to report Bonferroni corrected p-values instead of p-values, so each p-value is multiplied by the number of tests we ran and can by bigger than one, and its corresponding negative log p-value is therefore not lower-bounded by zero.

### Data availability

Our results can be found online in an interactive form: http://apps.chiragjpgroup.org/Aging_Biomarkers_Co-Dependencies/.

## Supplementary Material

Supplementary Figure

Supplementary Tables
