# Supplementary figures and images for: Age-dependent co-dependency structure of biomarkers in the general population of the United States
Source: Aging (Albany NY). 2019 Feb 28;11(5):1404–26. doi: 10.18632/aging.101842 (PMC6428110; doi:10.18632/aging.101842)

SUPPLEMENTARY FIGURE

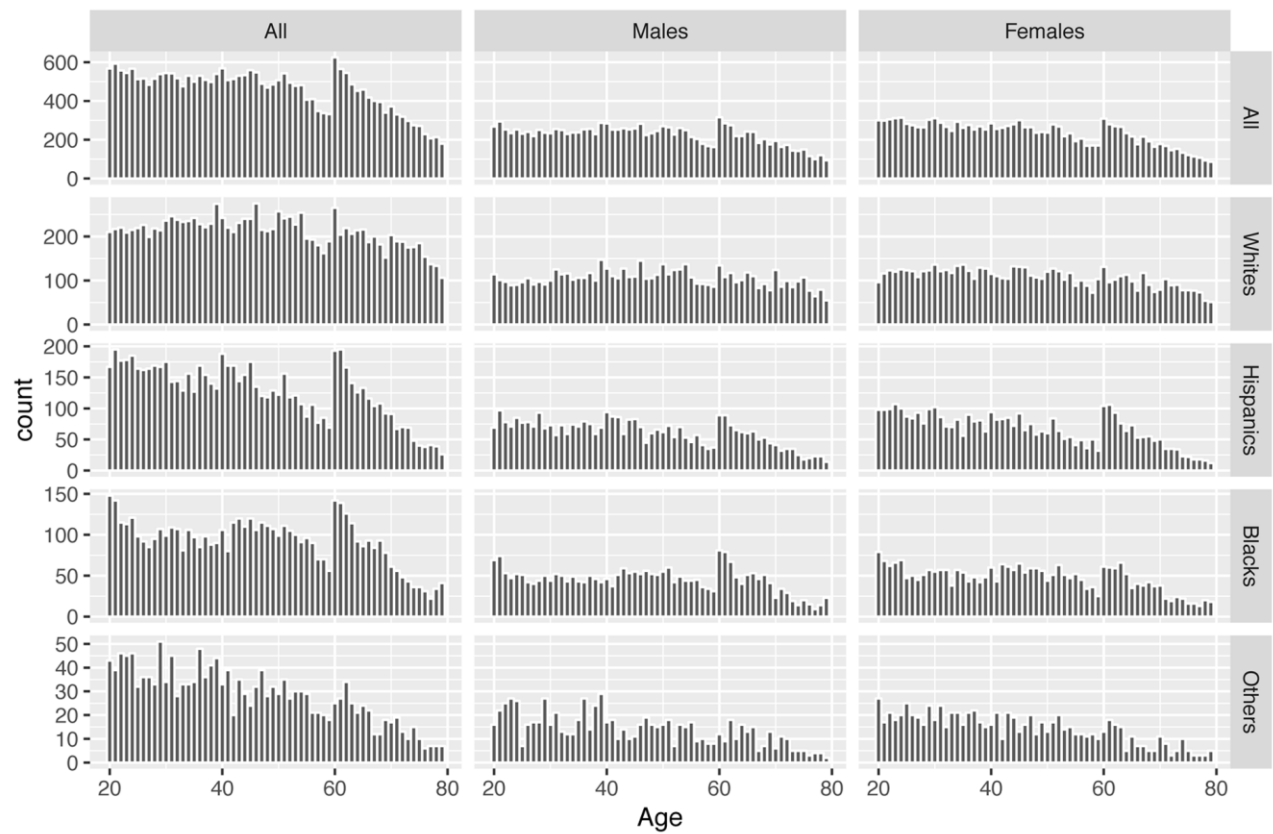

Figure S1. Age distribution in different demographic subgroups.

Supplement: Supplementary Figure [file aging-11-101842-s001.pdf]
