# Supplementary material for: Age-dependent co-dependency structure of biomarkers in the general population of the United States
Source: Aging (Albany NY). 2019 Feb 28;11(5):1404–26. doi: 10.18632/aging.101842 (PMC6428110; doi:10.18632/aging.101842)
Supplement: Supplementary Tables [file aging-11-101842-s002.pdf]

SUPPLEMENTARY TABLES

Table S1. Demographics of the UK Biobank dataset: sample sizes.

	All	Males	Females
All	502628	229168	273460
Prefer not to answer	1662	955	707
Do not know	217	110	107
White	571	327	244
Mixed	49	19	30
Asian or Asian British	43	20	23
Black or Black British	27	9	18
Chinese	1574	584	990
Other ethnic group	4560	1963	2597
British	442687	202371	240316
Irish	13213	6311	6902
Any other white background	16340	6293	10047
White and Black Caribbean	620	230	390
White and Black African	425	129	296
White and Asian	831	348	483
Any other mixed background	1033	379	654
Indian	5951	3012	2939
Pakistani	1837	1120	717
Bangladeshi	236	162	74
Any other Asian background	1815	980	835
Caribbean	4519	1647	2872
African	3396	1711	1685
Any other Black background	123	42	81

Table S2. Demographics of the UK Biobank dataset: age distribution.

	All	Males	Females
All	56.5 (50-63)	56.7 (50-64)	56.3 (50-63)
Prefer not to answer	56.7 (50-64)	57.3 (51-64)	55.9 (50-63)
Do not know	55.0 (48-62)	54.9 (47-62)	55.1 (48-62)
White	56.5 (50-63)	56.0 (49-63)	57.3 (52-63)
Mixed	55.4 (48-63)	55.6 (45-65)	55.2 (48-62)
Asian or Asian British	54.1 (45-62)	51.0 (42-55)	56.7 (52-64)
Black or Black British	53.6 (47-57)	55.6 (50-65)	52.7 (47-56)
Chinese	52.5 (46-58)	52.4 (45-59)	52.5 (46-58)
Other ethnic group	52.5 (46-59)	52.2 (45-59)	52.8 (46-59)
British	56.9 (51-63)	57.1 (51-64)	56.7 (50-63)
Irish	56.2 (50-63)	56.1 (49-63)	56.3 (50-63)
Any other white background	54.5 (47-61)	54.4 (47-61)	54.6 (47-62)
White and Black Caribbean	50.3 (44-55)	50.7 (44-57)	50.1 (44-54)
White and Black African	51.7 (45-58)	51.4 (45-57)	51.8 (45-58)
White and Asian	52.1 (44-59)	51.5 (44-58)	52.6 (45-60)
Any other mixed background	52.2 (45-59)	53.1 (45-61)	51.7 (45-58)
Indian	54.2 (47-61)	54.6 (47-62)	53.7 (47-60)
Pakistani	51.4 (44-57)	51.7 (44-57)	51.0 (44-57)
Bangladeshi	49.8 (42-55)	49.0 (42-54)	51.6 (44-59)
Any other Asian background	53.0 (45-60)	52.7 (45-60)	53.3 (46-60)
Caribbean	52.6 (46-59)	53.0 (46-59)	52.5 (46-58)
African	51.0 (44-57)	50.6 (44-56)	51.4 (45-57)
Any other Black background	51.8 (44-59)	51.7 (43-59)	51.9 (45-59)

The first number is the mean age of the demographic subgroup, the two numbers between parentheses are the 25th and 75th percentile of the age distribution.
